# Chest computed tomography in the diagnosis of COVID-19 in patients with false negative RT-PCR

**DOI:** 10.31744/einstein_journal/2021AO6363

**Published:** 2021-10-26

**Authors:** Eduardo Kaiser Ururahy Nunes Fonseca, Lorena Carneiro Ferreira, Bruna Melo Coelho Loureiro, Daniel Giunchetti Strabelli, Lucas de Pádua Gomes de Farias, Gabriel Abrantes de Queiroz, José Vitor Rassi Garcia, Renato de Freitas Teixeira, Victor Arcanjo Almeida Gama, Rodrigo Caruso Chate, Antonildes Nascimento Assunção, Márcio Valente Yamada Sawamura, Cesar Higa Nomura

**Affiliations:** 1 Hospital das Clínicas Faculdade de Medicina Universidade de São Paulo São PauloSP Brazil Hospital das Clínicas, Faculdade de Medicina, Universidade de São Paulo, São Paulo, SP, Brazil.

**Keywords:** COVID-19, Coronavirus, Coronavirus infections, Pneumonia, viral, Tomography, X-ray computed, Reverse transcriptase polymerase chain reaction

## Abstract

**Objective:**

To evaluate the role of chest computed tomography in patients with COVID-19 who presented initial negative result in reverse transcriptase-polymerase chain reaction (RT-PCR).

**Methods:**

A single-center, retrospective study that evaluated 39 patients with negative RT-PCR for COVID-19, who underwent chest computed tomography and had a final clinical or serological diagnosis of COVID-19. The visual tomographic classification was evaluated according to the Consensus of the Radiological Society of North America and software developed with artificial intelligence for automatic detection of findings and chance estimation of COVID-19.

**Results:**

In the visual tomographic analysis, only one of them (3%) presented computed tomography classified as negative, 69% were classified as typical and 28% as indeterminate. In the evaluation using the software, only four (about 10%) had a probability of COVID-19 <25%.

**Conclusion:**

Computed tomography can play an important role in management of suspected cases of COVID-19 with initial negative results in RT-PCR, especially considering those patients outside the ideal window for sample collection for RT-PCR.

## INTRODUCTION

In the context of highly infectious diseases, with the potential to spread quickly if the infected individuals are not properly oriented and isolated, the diagnosis must be made quick and accurate. Therefore, a diagnostic test should be practical and widely available, with good accuracy and fast results.

So far, unfortunately no laboratory and imaging tests for the diagnosis of the new coronavirus disease 2019 (COVID-19) meet all these characteristics.^(1-9)^ Hence, the use of several diagnostic methods, together with the clinical history, is the final form to make the diagnosis of infection by severe acute respiratory syndrome coronavirus 2 (SARS-CoV-2) in several cases.

Since the first cases of COVID-19 in December 2019, much has been learned about the role of diagnostic methods, and the limitations intrinsic to each method alone have been better described; for instance, it is known that the sensitivity of reverse transcriptase-polymerase chain reaction (RT-PCR) has high false-negative rates before 4 to 7 days after symptom onset.^(1-4,9-12)^ Moreover, chest tomography shows more false-negative results in up to 48 hours after the onset of symptoms, and the findings, although suggestive of COVID-19, can be found in several other diseases, including those of non-infectious origin. Therefore, the clinical correlation is essential.^(1,2,6,13-15)^

The first studies of pulmonary tomographic findings related to infection by the new coronavirus showed variable results of sensitivity and specificity compared to RT-PCR. However, these initial studies did not have a clear methodology on what would be considered positive in chest computed tomography (CT), and did not use any specific classification, which did not exist at the time.^(2,5,10,16,17)^ Only in March, with the pandemic already established, an American group related to the Radiological Society of North America (RSNA)^(18)^ published a classification of the main imaging findings of COVID-19.

Nonetheless, little has been discussed about the cases of negative RT-PCR and the role of CT, which can be a problem in the management of this group of patients.^(5)^ It is a fact that especially 2 weeks after the onset of symptoms,^(19,20)^ serological tests are of great value. However, there is a delay in obtaining the result of the test, which can go on for many days. In this context, CT can be of great help.

It is also noteworthy that there are currently software to aid making diagnosis by CT, which provide the probability of COVID-19 through the analysis of images with the help of artificial intelligence, in addition to automatically quantifying the extent of the disease, which can contribute to clinical decisions.^(21,22)^ But little has been studied about the accuracy of these software, especially in patients with negative RT-PCR.

## OBJECTIVE

To propose an initial assessment of the role of chest computed tomography in the diagnosis of cases with negative result in reverse transcriptase-polymerase chain reaction, although with a final diagnosis of COVID-19, using the Radiological Society of North America classification and an automatic software.

## METHODS

This is a retrospective observational study in patients referred to a tertiary reference hospital in Brazil, as suspected or probable cases of COVID-19, an initial negative RT-PCR, between March 16, 2020 and May 13, 2020. The Ethics Committee approved the waiver of informed consent.

The inclusion criteria were based on the definition of cases by the World Health Organization (WHO).^(23)^ Also, patients should have a first negative result in RT-PCR for COVID-19 and a definitive clinical diagnosis at the end of the study (May 13, 2020).

Exclusion criteria were patients who had not been tested or had no results available for RT-PCR test for SARS-CoV-2 virus; patients who had not undergone chest CT; presence of an important movement artifact on chest CT; initial positive result in RT-PCR test for SARS-CoV-2; absence of a definitive clinical diagnosis; final alternative clinical or serological diagnosis; and lack of clear clinical information about duration of symptoms.

Study carried out at the *Hospital das Clínicas da Faculdade de Medicina da Universidade de São Paulo*. This study was approved by the Research Ethical Committee of the organization, under protocol 4.237.616, CAAE: 32226920.2.0000.0068.

### Computed tomography

**Figure 1:**
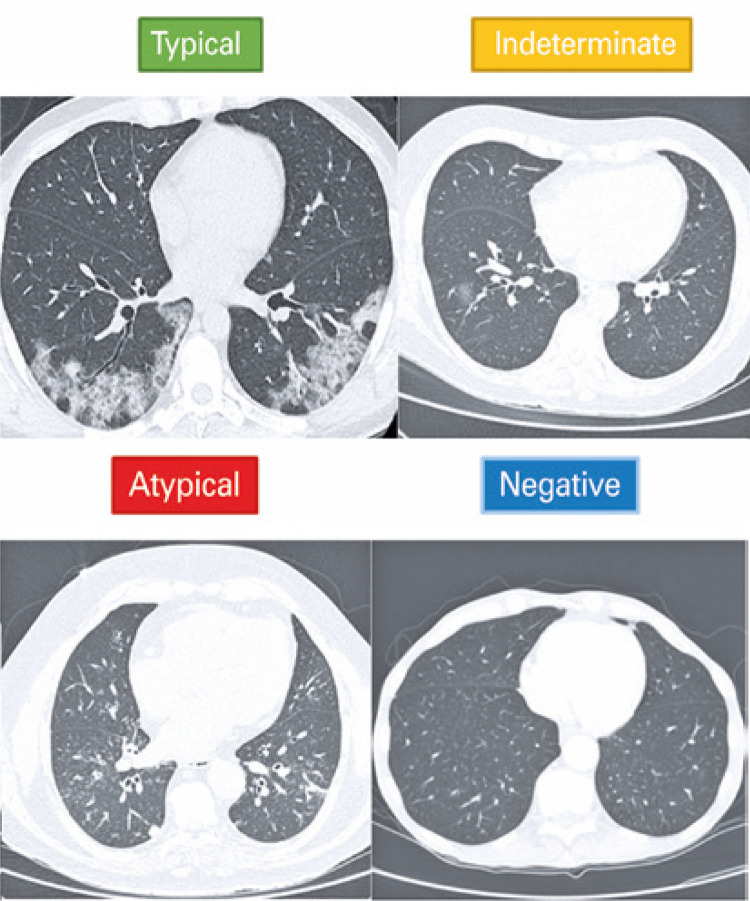
Illustrative cases based on the Radiological Society of North America(18) Consensus classification. Unenhanced chest computed tomography axial images of the lungs showing, from left to right, typical appearance for COVID-19 (peripheral bilateral ground-glass opacities); indeterminate appearance for COVID-19 (isolated and unilateral ground-glass opacity); atypical appearance for COVID-19 (bronchial wall thickening associated with centrilobular “tree-in-bud” nodules); and negative for pneumonia

Chest CT images obtained from CT scanners with 64 to 320-detector rows of were evaluated. All examinations were performed in the supine position, during maximal inspiration, with no use of contrast medium. The acquisition parameters included voltage 80kVp to 120kVp, tube current of 10mA to 440mA, both varying according to institutional protocols already established for each device and biotype of the patient, and reconstruction with thickness ranging between 1mm and 1.5mm. The images were evaluated within a set of more than 600 cases referred to our service with suspected COVID-19, by two radiologists with two-year experience in chest imaging. Initially, the evaluators did not have access to results of RT-PCR. All patients were categorized based on the RSNA^(18)^ Consensus classification (typical, indeterminate, atypical or negative for pneumonia – Figure 1). Disagreement cases were decided by consensus between the two radiologists. The typical pattern of the RSNA was considered as a positive CT scan for COVID-19. The other patterns were considered as not suggestive of COVID-19. Simultaneously, these cases were evaluated using the Huawei Cloud (Hong Kong, CN, China) - AI-Assisted Diagnosis for COVID-19 software. The probability of 90% or more was considered suggestive of infection COVID-19. Percentages <25% were considered non-suggestive of COVID-19. Situations between 25% and 90% or diagnosed as severe pneumonia were considered as indeterminate. After the blind tomographic analysis, an analysis of the electronic medical records of all selected patients was carried out, searching for duration of symptoms, RT-PCR and serology results, and final clinical diagnosis.

### Statistical analysis

Statistical analysis was performed using Prism (GraphPad Software, Inc., San Diego, CA, USA). Data were described using means with standard deviation for quantitative variables, and through frequency values for qualitative variables (both in relative and absolute values). Fisher exact test was applied to compare categorical variables and the Student’s *t* test, to compare means. To quantify the inter-observer agreement, Fleiss Kappa was calculated among observers. Interobserver agreement was considered weak for a Kappa value of 0.01 to 0.20, reasonable for 0.21 to 0.40, moderate for 0.41 to 0.60, substantial for 0.61 to 0.80, and almost perfect for 0.81 to 1.00.

## RESULTS

**Figure 2:**
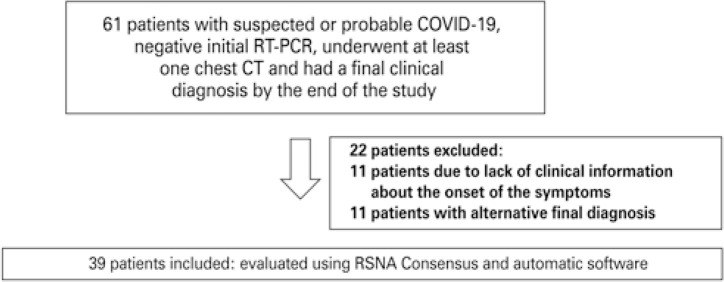
Flow diagram outlining both the inclusion and exclusion criteria of the study RT-PCR: reverse transcriptase-polymerase chain reaction; CT: computed tomography; RSNA: Radiological Society of North America.

A total of 61 patients met the inclusion criteria. After applying the exclusion criteria, 39 patients remained and had the RSNA classification evaluated and compared. These same patients were also evaluated by the software. Figure 2 illustrates this process.

The mean time from onset of symptoms to RT-PCR was 7.67±3.5 days and the mean time from onset of symptoms until CT was 7.92±4.0 days, with no statistically significant difference between them (p=0.7619).

### Classification as per the criteria of the Radiological Society of North America

Of the 39 patients, 27 (69%) were classified as typical, 11 as indeterminate (28%) and one as negative (3%). There were no patients classified as atypical appearance. There was an almost perfect agreement in the application of the RSNA Consensus (97%), with only one case mentioned as typical by one examiner and indeterminate by another, which determined a Kappa index of 0.95 (95% confidence interval – 95%CI: 0.833 to 1,000). This case was classified as typical by consensus.

The mean time from the onset of symptoms to the performance of RT-PCR and CT is shown in table 1.

**Table 1:** Time between the onset of symptoms until reverse transcriptase-polymerase chain reaction and computed tomography as per the Radiological Society of North America classification

RSNA Consensus	RT-PCR	CT
Typical	6.67±2.8	6.81±2.9
Indeterminate	9.91±4.1	10.45±5.2
p value*	0.0078*	0.0087*

Results expressed as means±standard deviation.

* p<0.05 was considered statistically significant.

RSNA: Radiological Society of North America; RT-PCR: reverse transcriptase-polymerase chain reaction; CT: computed tomography.

There was one patient classified as negative who underwent CT and RT-PCR with 10 days of symptoms and was not included in the table alone.

### Evaluation by software

A similar evaluation was made for 39 patients considering the probability of COVID-19 by the software, as shown in table 2.

**Table 2:** Mean time from onset of symptoms until reverse transcriptase-polymerase chain reaction and computed tomography by the software

Software classification	RT-PCR	TC
COVID-19 probability >90%	7.2±3.6	7.4±4.3
COVID-19 probability <90%	8.2±3.4	8.5±3.6
p value*	0.3977	0.4033

Results expressed as means±standard deviation.

* p<0.05 was considered statistically significant.

RT-PCR: reverse transcriptase-polymerase chain reaction; CT: computed tomography.

Of the 39 patients, 20 (51.3%) were classified as a probability of COVID-19 >90% and, of the others, 11 (28.2%) as a probability <90%, and eight as a high probability of severe pneumonia (20.5%). It stood out that only four patients (approximately 10%) showed probabilities of COVID-19 less than 25%, one of them also classified as negative by the RSNA, and the others classified as indeterminate by the RSNA. Eight patients were classified as as possible severe pneumonia as the most likely diagnosis, a definition applied by the software for those exams with >70% involvement of the lung parenchyma.

The agreement between the RSNA Consensus and the software was also evaluated, which resulted in a Kappa index of 0.43 (95%CI: 0.17-0.70). It is remarkable that, of the patients classified as indeterminate, two of them were identified as a chance of COVID-19 by the software, while five patients with a typical pattern by the RSNA were classified as a COVID-19 probability <90%. Of those patients classified as as possible severe pneumonia by the software, four were classified as indeterminate by RSNA and four as typical.

## DISCUSSION

An accurate and rapid diagnosis is essential in managing cases of a highly infectious disease to mitigate dissemination as much as possible. The gold standard for diagnosis of COVID-19 is both RT-PCR or serology. However, both tests have limitations, essentially related to the onset of symptoms.^(1,3,4,10,19,20)^

Computed tomography can help prevent possible cases of COVID-19 from being lost due to RT-PCR false-negative results. However, given the non-specificity of tomographic patterns, the findings must be critically evaluated together with clinical data. Moreover, any patterns of findings must be interpreted as to the likelihood of representing pulmonary involvement by COVID-19 or not. Thus, in March 2020, an RSNA Consensus was published.^(18)^ This classification proved to be practical for use in the pandemic to guide the tomographic evaluation of COVID-19. In addition, some studies have already shown that its application in the context of the pandemic, especially considering the typical appearance, leads to good values of sensitivity and specificity.^(24,25)^

Furthermore, a software was developed to aid making diagnosis based on imaging tests, which provides a probability of tomographic findings represent pulmonary involvement by COVID-19, and quantifies the involvement of the lungs.^(21,22,26,27)^

There is still little information about the role of imaging in patients with negative RT-PCR, but who are suspected or probable cases of COVID-19.^(5,14,28)^ Thus, the present study evaluated and compared the performance of the visual tomographic classification of the RSNA and automatic software, using information of patients upon admission, who despite their initial negative RT-PCR, had their final clinical or laboratory diagnosis of COVID-19.

Our results demonstrate a good overall ability of the CT to detect alterations in this group: of 39 patients who were evaluated in the RSNA classification,^(18)^ only one of them had no CT findings. It should also be noted that there was no case considered atypical, an appearance that has already been demonstrated^(25)^ as the most related to a diagnosis other than COVID-19.

Considering the automatic software, only four patients out of 39 had less than 25% probability of having COVID-19, one of which was also considered negative by the RSNA Consensus. Considering such findings, CT, either through the RSNA Consensus or through the software, detected changes in about 90% of these patients. Thus, it may be interesting to use the software as a screening for exams, so that those patients above a certain risk probability of COVID-19 could be flagged to be evaluated first within the image analysis system.

Another point to be highlighted was that almost 70% of these patients had a typical CT scan for COVID-19 already in the first exam using the RSNA classification, a rapid assessment, performed in less than 10 minutes, and more than half was classified as a probability of COVID-19 >90% by the software, which occurs almost instantly.

Given these findings, CT may provide good initial guidance for patients with clinical symptoms of flu-like syndrome before the result of RT-PCR, and even after an initial negative result.^(15)^ Besides, in places with no radiologists available, the use of the software can provide an interesting immediate aid, even patients with negative RT-PCR.

Considering the distribution of the RSNA Consensus patterns with the mean time of symptoms, it was longer in the indeterminate group (approximately 10 days) compared to the typical pattern. Possibly, it is explained by patients with more extensive involvement by the disease, perhaps reflecting the pattern of severe respiratory distress syndrome, in which the typical rounded or peripheral multifocal pattern of pulmonary opacities is replaced by more diffuse findings, consequently classified as indeterminate. These findings are under the temporal evolutionary pattern of COVID-19 tomographic findings, which tend to peak between 9 to 13 days.^(15,29)^ Thus, perhaps more extensive findings should be considered more suggestive of COVID-19, even in cases with an indeterminate appearance, especially in those patients with longer period of symptoms.

There were no differences between the mean time of symptoms between those patients classified as >90% chance of COVID-19 by the software and those classified as <90% chance of COVID-19, although the mean time from onset of symptoms was longer in patients classified as possible severe pneumonia, despite the absence of statistical significance, most likely reflecting the same mechanism. We also emphasize that there were no significant differences in the time elapsed between symptoms between the performance of RT-PCR and CT, a factor that could act as a confounder.

There is also an excellent interobserver correlation for the classification of RSNA in these patients, similar to what has been observed in the literature for differentiating patients with and without COVID-19 in the general population.^(24,25)^ This corroborates its good reproducibility, a crucial factor in the context of a pandemic. However, we emphasize that, although no specific training has taken place, both examiners are chest radiologists who have been working since the beginning of the pandemic with chest CT in patients suspected of COVID-19.

Our study has some limitations that must be considered. The retrospective analysis methodology must be considered. Also, the initially small number of patients may have partially influenced the analyses. However, we decided to proceed with this initial analysis due to the great percentage of suggestive tomographic findings in these patients with negative RT-PCR, both by the RSNA classification and the software; these findings can certainly influence in the management of patients in the context of the pandemic. The present study was performed in a tertiary center that only received patients with clinical suspicion of COVID-19 during the months of the study, so that the pretest probability of COVID-19 was possibly increased. Furthermore, we used the final clinical diagnosis as a reclassification criterion, which may have led to some false-positive results. However, we believe that this approach reflects the practical management of most patients in the pandemic, which has been used in large trials.^(30-32)^ Yet, in the current pandemic context, the chance of false positive results by clinical evaluation is certainly smaller.

## CONCLUSION

We concluded computed tomography may have an important role in the management of suspected cases of COVID-19, who presented an initial negative result in reverse transcriptase-polymerase chain reaction, especially considering the patients outside the ideal sample collection window. Only one patient had an initial negative computed tomography according to the Radiological Society of North America Consensus classification, and the software demonstrated probability of COVID-19 >25% in most patients, who had initial negative result in reverse transcriptase-polymerase chain reaction, but had a final diagnosis of COVID-19.
