# Supplementary material for: Chest computed tomography in the diagnosis of COVID-19 in patients with false negative RT-PCR
Source: Einstein (Sao Paulo). 2021 Oct 26;19:eAO6363. doi: 10.31744/einstein_journal/2021AO6363 (PMC8555875; doi:10.31744/einstein_journal/2021AO6363)
Supplement: Translation

# Translation: Tomografia computadorizada de tórax no diagnóstico de COVID-19 em pacientes com resultado falso-negativo na RT-PCR

**Authors:** Eduardo Kaiser Ururahy Nunes Fonseca, Lorena Carneiro Ferreira, Bruna Melo Coelho Loureiro, Daniel Giunchetti Strabelli, Lucas de Pádua Gomes de Farias, Gabriel Abrantes de Queiroz, José Vitor Rassi Garcia, Renato de Freitas Teixeira, Victor Arcanjo Almeida Gama, Rodrigo Caruso Chate, Antonildes Nascimento Assunção, Márcio Valente Yamada Sawamura, Cesar Higa Nomura

**Affiliations:** 1 Brasil Hospital das Clínicas, Faculdade de Medicina, Universidade de São Paulo, São Paulo, SP, Brasil.

**Keywords:** COVID-19, Coronavírus, Infecções por coronavírus, Pneumonia viral, Tomografia computadorizada por raios X, Reação em cadeia da polimerase via transcriptase reversa

## Abstract

**Objetivo:**

Avaliar o papel da tomografia computadorizada de tórax em pacientes com COVID-19 que apresentaram reação em cadeia da polimerase via transcriptase reversa (RT-PCR) inicial falsamente negativa.

**Métodos:**

Estudo retrospectivo de centro único que avaliou 39 pacientes com RT-PCR negativa para COVID-19, submetidos à tomografia computadorizada de tórax e que tiveram diagnóstico final clínico ou serológico de COVID-19. A classificação tomográfica visual foi avaliada de acordo com o Consenso da *Radiological Society of North America* e *o* software desenvolvido com inteligência artificial para detecção automática de achados e estimativa de probabilidade de COVID-19.

**Resultados:**

Na análise tomográfica visual, somente um deles (3%) apresentou tomografia computadorizada classificada como tendo resultado negativo, 69% foram classificados como típicos e 28% como indeterminados. Na avaliação com uso *de* software*,* somente quatro (cerca de 10%) tiveram probabilidade de COVID-19 <25%.

**Conclusão:**

A tomografia computadorizada pode desempenhar papel importante no manejo de casos suspeitos de COVID-19 com RT-PCR inicialmente negativa, principalmente levando-se em consideração os pacientes que estão fora da janela ideal para coleta de amostra para RT-PCR.

## INTRODUÇÃO

No contexto das doenças altamente infecciosas, com potencial de se disseminarem rapidamente se os infectados não forem devidamente orientados e isolados, o diagnóstico precisa ser rápido e preciso. Assim um teste diagnóstico deve ser prático e estar amplamente disponível, com boa precisão e resultados rápidos.

Infelizmente, ao menos por enquanto, nenhum dos exames laboratoriais ou de imagem para o diagnóstico da doença pelo coronavírus 2019 (COVID-19) apresenta todas essas características.^(1-9)^ O uso de diversos métodos diagnósticos, junto da história clínica, é a forma final de diagnóstico da infecção pelo coronavírus da síndrome respiratória aguda grave 2 (SARS-CoV-2) em vários casos.

Desde os primeiros casos de COVID-19, em dezembro de 2019, muito se aprendeu sobre o papel dos métodos diagnósticos, de modo que as limitações intrínsecas de cada método isoladamente foram mais bem descritas. Por exemplo: sabe-se que a sensibilidade da reação em cadeia da polimerase via transcriptase reversa (RT-PCR) tem altas taxas de falso-negativo antes de um período de 4 a 7 dias após o início dos sintomas.^(1-4,9-12)^ Além disso, a tomografia de tórax mostra mais resultados falso-negativos em até 48 horas após o início dos sintomas, e os achados, embora sugestivos de COVID-19, podem ser encontrados em várias outras doenças, inclusive as de origem não infecciosa. Desse modo, a correlação com a clínica é essencial.^(1,2,6,13-15)^

Os primeiros estudos sobre os achados tomográficos pulmonares relacionados à infecção pelo novo coronavírus mostraram resultados variáveis de sensibilidade e especificidade em relação à RT-PCR. No entanto, tais estudos iniciais não tinham metodologia clara sobre o que seria considerado resultado positivo numa tomografia computadorizada (TC) de tórax e nem utilizavam uma classificação específica, inexistente na época.^(2,5,10,16,17)^ Somente em março, já com a pandemia estabelecida, um grupo americano, vinculado a *Radiological Society of North America* (RSNA)^(18)^ publicou uma classificação dos principais achados em exames de imagem da COVID-19.

Entretanto, pouco se discutiu sobre os casos de testes de RT-PCR negativos e o papel da TC, o que pode ser um problema no manejo desse grupo de pacientes.^(5)^ É fato que, principalmente 2 semanas após o início dos sintomas,^(19,20)^ os testes sorológicos são de grande valia. Porém há demora na obtenção dos resultados do teste, que pode se estender por muitos dias. Nesse contexto, a TC pode ser de grande valia.

Ressalta-se também que, atualmente, existem programas de auxílio diagnóstico à TC, que fornecem a probabilidade de COVID-19 por meio da análise de imagens com auxílio de inteligência artificial, além de quantificar automaticamente a extensão da doença, o que pode contribuir para as decisões clínicas.^(21,22)^ No entanto, pouco se estudou sobre a precisão desses programas, principalmente em pacientes com RT-PCR negativa.

## OBJETIVO

Propor uma avaliação inicial do papel da tomografia computadorizada de tórax no diagnóstico de casos com resultado negativo no teste de reação em cadeia da polimerase via transcriptase reversa, embora com diagnóstico final de COVID-19, utilizando a classificação da *Radiological Society of North America* e um software automático.

## MÉTODOS

Este é um estudo observacional retrospectivo realizado com pacientes encaminhados a um hospital terciário de referência no Brasil como casos suspeitos ou prováveis de COVID-19, com exame inicial de RT-PCR negativos, entre 16 de março de 2020 e 13 de maio de 2020. O Comitê de Ética aprovou a dispensa do Termo de Consentimento Livre e Esclarecido dos pacientes.

Os critérios de inclusão foram baseados na definição de casos divulgada pela Organização Mundial da Saúde (OMS).^(23)^ Além disso, os pacientes deveriam ter resultado inicial negativo de RT-PCR para COVID-19 e um diagnóstico clínico final definido ao término do estudo (13 de maio de 2020).

Os critérios de exclusão foram pacientes que não foram testados ou não tinham resultados disponíveis da RT-PCR para SARS-CoV-2; não foram submetidos à TC de tórax; com presença de um artefato de movimento importante na TC de tórax; com resultado inicial positivo no teste da RT-PCR para SARS-CoV-2; com ausência de diagnóstico clínico definitivo; com diagnóstico final clínico ou sorológico alternativo e com ausência de informações clínicas claras sobre a duração dos sintomas.

Estudo realizado no Hospital das Clínicas da Faculdade de Medicina da Universidade de São Paulo. Este estudo foi aprovado pelo Comitê de Ética em Pesquisa da instituição, com o protocolo 4.237.616, CAAE: 32226920.2.0000.0068.

### Tomografia computadorizada

Foram avaliadas imagens de TC de tórax obtidas de tomógrafos com 64 a 320 detectores. Todos os exames foram realizados em decúbito dorsal, em inspiração máxima, sem uso de meio de contraste. Os parâmetros de aquisição incluíram voltagem de 80kVp a 120kVp, corrente do tubo de 10mA a 440mA (ambas variando de acordo com os protocolos institucionais já estabelecidos para cada dispositivo e biotipo dos pacientes) e reconstrução com espessura variando entre 1mm e 1,5mm. As imagens foram avaliadas dentro de uma série de mais de 600 casos encaminhados ao nosso serviço com suspeita de COVID-19, por dois radiologistas com 2 anos de experiência em radiologia torácica. Os avaliadores não tiveram acesso inicialmente aos resultados da RT-PCR. Todos os pacientes foram categorizados com base na classificação do Consenso da RSNA^(18)^ (padrões típico, indeterminado, atípico ou negativo para pneumonia – Figura 1). Os casos de discordância foram decididos por consenso entre os dois radiologistas. Uma TC com o padrão típico do RSNA foi considerada positiva para COVID-19. Os demais padrões foram considerados não sugestivos de COVID-19. Simultaneamente, esses casos foram avaliados por meio do software Huawei Cloud (Hong Kong, CN, China) *AI-Assisted Diagnosis for* COVID-19. A probabilidade de 90% ou mais foi considerada sugestiva de infecção por COVID-19. As percentagens <25% foram consideradas não sugestivas de COVID-19. As situações entre 25% e 90% ou com diagnóstico de pneumonia grave foram consideradas indeterminadas. Após a análise tomográfica às cegas, foi realizada a análise dos prontuários eletrônicos de todos os pacientes selecionados, sendo feita uma pesquisa sobre duração dos sintomas, os resultados da RT-PCR e da sorologia e o diagnóstico clínico final.

**Figura 1:** Casos ilustrativos baseados na classificação do Consenso da *Radiological Society of North America*.(18) Imagens axiais de tomografia computadorizada de tórax sem contraste, mostrando pulmões com padrão, da esquerda para a direita, típico de COVID-19 (opacidades em vidro fosco periféricas bilaterais); indeterminado para COVID-19 (opacidade em vidro fosco isolada e unilateral); atípico para COVID-19 (espessamento da parede brônquica associado a nódulos centrolobulares em “árvore em brotamento”); negativo para pneumonia

### Análise estatística

A análise estatística foi realizada por meio do programa Prism (GraphPad Software, Inc., San Diego, CA, Estados Unidos). Os dados foram descritos usando médias com desvio-padrão para as variáveis quantitativas e por meio de valores de frequência para as variáveis qualitativas (tanto em valores relativos quanto absolutos). Foram aplicados o teste exato de Fisher, para comparar as variáveis categóricas, e o teste *t* de Student, para comparar as médias. Para quantificar a concordância interobservadores, foi calculado o Kappa de Fleiss entre os observadores. A concordância interobservadores foi considerada fraca para um valor de Kappa de 0,01 a 0,20, razoável para 0,21 a 0,40, moderada para 0,41 a 0,60, substancial para 0,61 a 0,80 e quase perfeita para 0,81 a 1,00.

## RESULTADOS

Atenderam aos critérios de inclusão 61 pacientes. Depois de serem aplicados os critérios de exclusão, 39 pacientes permaneceram na amostra e tiveram sua classificação RSNA avaliada e comparada. Esses mesmos pacientes também foram analisados pelo software. A figura 2 ilustra esse processo.

**Figura 2:** Fluxograma mostrando os critérios de inclusão e exclusão do estudo RT-PCR: reação em cadeia da polimerase via transcriptase reversa; TC: tomografia computadorizada; RSNA: *Radiological Society of North America*.

O tempo médio do início dos sintomas até a RT-PCR foi de 7,67±3,5 dias, e o tempo médio desde os sintomas até a TC foi de 7,92±4,0 dias, sem diferença estatisticamente significativa entre eles (p=0,7619).

### Classificação de acordo com os critérios da *Radiological Society of North America*

Dos 39 pacientes, 27 (69%) foram classificados como típicos, 11 como indeterminados (28%) e um como negativo (3%). Não houve pacientes classificados como tendo um padrão atípico. Houve concordância quase perfeita na aplicação do Consenso da RSNA (97%), com um único caso mencionado como típico por um examinador e indeterminado por outro, o que determinou um índice Kappa de 0,95 (intervalo de confiança 95% – IC95%: 0,833 para 1.000). Esse caso foi classificado como típico por consenso.

O tempo médio desde o início dos sintomas até a realização da RT-PCR e da TC está mostrado na tabela 1.

**Tabela 1:** Tempo entre os sintomas até a reação em cadeia da polimerase via transcriptase reversa e a tomografia computadorizada, pela classificação da *Radiological Society of North America*

Consenso da RSNA	RT-PCR	TC
Típico	6,67±2,8	6,81±2,9
Indeterminado	9,91±4,1	10,45±5,2
Valor de p*	0,0078*	0,0087*

Resultados expressos por média±desvio-padrão.

* Um valor de p<0,05 foi considerado estatisticamente significativo.

RSNA: *Radiological Society of North America;* RT-PCR: reação em cadeia da polimerase via transcriptase reversa; TC: tomografia computadorizada.

Houve um paciente classificado como negativo que foi submetido à TC e à RT-PCR com 10 dias de sintomas e não foi incluído na tabela de forma isolada.

### Avaliação por software

Uma avaliação semelhante foi realizada para os 39 pacientes cuja probabilidade de COVID-19 foi examinada pelo software de análise, conforme mostrado na tabela 2.

**Tabela 2:** Tempo médio desde o início dos sintomas até reação em cadeia da polimerase via transcriptase reversa e tomografia computadorizada pelo software

Classificação pelo software	RT-PCR	TC
Probabilidade de COVID-19 >90%	7,2±3,6	7,4±4,3
Probabilidade de COVID-19 <90%	8,2±3,4	8,5±3,6
Valor de p*	0,3977	0,4033

Resultados expressos por média±desvio-padrão.

* Um valor de p<0,05 foi considerado estatisticamente significativo.

RT-PCR: reação em cadeia da polimerase via transcriptase reversa; TC: tomografia computadorizada.

Dos 39 pacientes, 20 (51,3%) foram classificados como probabilidade >90% de COVID-19 e, dos demais, 11 (28,2%) como probabilidade <90% e oito como probabilidade alta de pneumonia grave (20,5%). Ressalta-se, porém, que apenas quatro pacientes (cerca de 10%) apresentaram probabilidade de COVID-19 menor que 25%, sendo um deles também classificado como negativo pelo RSNA; os demais foram classificados como indeterminado pelo RSNA. Oito pacientes foram classificados com possível pneumonia grave como diagnóstico mais provável, definição aplicada pelo software para os exames com >70% de envolvimento do parênquima pulmonar.

A concordância entre o Consenso RSNA e o software também foi avaliada, o que resultou em um índice Kappa de 0,43 (IC95%: 0,17-0,70). Dos pacientes classificados como indeterminados, dois deles foram identificados como tendo probabilidade de COVID-19 pelo software, ao passo que cinco pacientes com padrão típico pelo RSNA foram classificados como tendo probabilidade <90% de COVID-19. Dos pacientes classificados como possível pneumonia grave pelo software, quatro foram classificados como indeterminados pelo RSNA e quatro como típicos.

## DISCUSSÃO

Um diagnóstico preciso e rápido é essencial no manejo de casos de uma doença altamente infecciosa, para mitigar tanto quanto possível o contágio. O padrão-ouro para o diagnóstico de COVID-19 é a RT-PCR, além da sorologia. No entanto, ambos os testes apresentam limitações, essencialmente relacionadas ao tempo de início dos sintomas.^(1,3,4,10,19,20)^

A TC pode ajudar a prevenir que possíveis casos de COVID-19 sejam perdidos devido a resultados falso-negativos da RT-PCR. No entanto, dada a não especificidade dos padrões tomográficos, os achados devem ser avaliados criticamente, em conjunto com os dados clínicos. Ainda, quaisquer padrões de achados devem ser interpretados quanto à probabilidade de representar envolvimento pulmonar pela COVID-19 ou não. Assim, em março de 2020, foi publicado um Consenso da RSNA.^(18)^ Essa classificação mostrou-se prática para uso na pandemia, orientando a avaliação tomográfica da COVID-19. Além disso, alguns estudos já demonstraram que sua aplicação no contexto da pandemia, principalmente em relação ao padrão típico, resulta em bons valores de sensibilidade e especificidade.^(24,25)^

Também foi desenvolvido um software para auxiliar no diagnóstico por imagem, que fornece uma probabilidade de os achados tomográficos representarem envolvimento pulmonar pela COVID-19 e quantifica o envolvimento dos pulmões.^(21,22,26,27)^

Ainda há poucas informações sobre o papel dos exames de imagem nos pacientes com RT-PCR negativa, mas que constituem casos suspeitos ou prováveis de COVID-19.^(5,14,28)^ Assim, o presente estudo avaliou e comparou o desempenho da classificação tomográfica visual do RSNA e do software automático, que utiliza a admissão de pacientes que, apesar da RT-PCR inicial com resultado negativo, tiveram a COVID-19 como diagnóstico final clínico ou laboratorial.

Nossos resultados demonstram boa capacidade geral da TC em detectar alterações nesse grupo: dos 39 pacientes avaliados na classificação RSNA,^(18)^ apenas um deles não apresentou achados tomográficos. Ressalta-se, também, que não houve nenhum caso considerado atípico, padrão esse que já foi demonstrado^(25)^ ser o mais relacionado a outro diagnóstico que não ao de COVID-19.

Em relação ao software automático, apenas quatro pacientes, de 39, tiveram menos de 25% de chance de ter COVID-19, sendo que um deles também foi considerado negativo pelo Consenso RSNA. Diante de tais achados, a TC, seja pelo Consenso RSNA ou pelo software, detectou alterações em cerca de 90% desses pacientes. Assim, pode ser interessante utilizar o software como triagem para exames, de forma que os pacientes que estiverem acima de uma determinada probabilidade de risco de COVID-19 sejam sinalizados para serem avaliados primeiro no sistema de análise de imagens.

Outro ponto a ser destacado foi o de que quase 70% desses pacientes apresentavam TC típica para COVID-19 já no primeiro exame pela classificação RSNA, que é uma avaliação rápida, realizada em menos de 10 minutos, e que mais da metade deles foi classificada como tendo probabilidade de COVID-19 >90% pelo software, o que ocorre quase que instantaneamente.

Diante desses achados, a TC pode fornecer uma boa orientação inicial para pacientes com sintomas clínicos de síndrome gripal antes do resultado da RT-PCR e mesmo após resultado inicial negativo.^(15)^ Além disso, em locais sem radiologistas disponíveis, a utilização do software pode fornecer ajuda imediata interessante, mesmo em pacientes com RT-PCR negativa.

Considerando a distribuição dos padrões de Consenso da RSNA com o tempo médio de sintomas, foi maior no grupo indeterminado (cerca de 10 dias) em relação ao padrão típico. Possivelmente, isso seja explicado por ter havido pacientes com envolvimento mais extenso pela doença, talvez refletindo o padrão da síndrome do desconforto respiratório grave, em que o padrão arredondado ou multifocal periférico típico das opacidades pulmonares é substituído por achados mais difusos, sendo consequentemente classificado como indeterminado. Esses achados estão de acordo com o padrão evolutivo temporal dos achados tomográficos da COVID-19, que tendem a ter pico entre 9 e 13 dias.^(15,29)^ Assim, talvez achados mais extensos devam ser considerados mais sugestivos de COVID-19, mesmo em casos que apresentem padrão indeterminado, especialmente naqueles pacientes com maior tempo de sintomas.

Não houve diferenças entre o tempo médio de sintomas entre os pacientes classificados como tendo probabilidade >90% de COVID-19 pelo software e aqueles classificados como tendo probabilidade <90% de COVID-19, embora o tempo médio de início dos sintomas tenha sido maior nos classificados como tendo possível pneumonia grave, apesar da ausência de significância estatística, muito provavelmente refletindo o mesmo mecanismo. Ressalta-se também que não houve diferenças significativas no tempo decorrido entre os sintomas e a realização da RT-PCR e da TC, o que pode ter atuado como fator de confusão.

Também existe excelente correlação interobservador na classificação do RSNA nesses pacientes, semelhante ao que tem sido observado na literatura para diferenciação de pacientes com e sem COVID-19 na população em geral.^(24,25)^ Isso corrobora sua boa reprodutibilidade, um fator crucial no contexto de uma pandemia. No entanto, destaca-se que, embora não tenha sido realizado treinamento específico, ambos os examinadores eram radiologistas torácicos que atuavam desde o início da pandemia com TC de tórax em pacientes com suspeita de COVID-19.

Este estudo apresenta algumas limitações que devem ser levadas em consideração. A metodologia da análise retrospectiva é uma delas. Além disso, o número inicialmente pequeno de pacientes pode ter influenciado parcialmente nas análises. No entanto, optou-se por prosseguir com essa análise inicial, devido ao grande percentual de achados tomográficos sugestivos nos pacientes com RT-PCR negativa, tanto pela classificação RSNA quanto pelo software; esses achados certamente podem influenciar no manejo dos pacientes nesse contexto da pandemia. O presente estudo foi realizado em um centro terciário que só recebeu pacientes com suspeita clínica de COVID-19 durante os meses do estudo, de modo que a probabilidade pré-teste de COVID-19 possivelmente aumentou. Além disso, utilizou-se o diagnóstico clínico final como critério de reclassificação, o que pode ter levado a alguns resultados falso-positivos. Porém acredita-se que essa abordagem reflete o manejo prático da maioria dos pacientes na pandemia, que tem sido feito em grandes estudos.^(30-32)^ Além disso, no atual contexto pandêmico, a probabilidade de falso-positivos pela avaliação clínica é certamente menor.

## CONCLUSÃO

A tomografia computadorizada pode ter papel importante no manejo de casos suspeitos de COVID-19, que apresentaram resultado negativo inicial na reação em cadeia da polimerase via transcriptase reversa, principalmente levando em consideração os pacientes que estão fora da janela ideal de coleta de amostras. Somente um paciente teve resultado negativo na tomografia computadorizada inicial, de acordo com a classificação do Consenso da *Radiological Society of North America*, e o software demonstrou probabilidade de COVID-19 >25% na maioria dos pacientes que apresentaram resultado inicial negativo na reação em cadeia da polimerase via transcriptase reversa, mas tiveram diagnóstico final de COVID-19.
